# Skill adaption in sport and movement: Practice design considerations for 360° VR

**DOI:** 10.3389/fpsyg.2023.1124530

**Published:** 2023-02-17

**Authors:** Riki Lindsay, Sharna Spittle, Michael Spittle

**Affiliations:** ^1^Institute of Education, Arts and Community, Federation University, Ballarat, VI, Australia; ^2^College of Sport and Exercise Science, Victoria University, Footscray, VI, Australia; ^3^Institute for Health and Sport, Victoria University, Footscray, VI, Australia

**Keywords:** 360°VR, immersive video, sport psychology, virtual reality, neuroscience

## 1. Introduction

Imagine being a gymnast wanting to develop a new routine. What could you do? One option would be to observe someone else performing the routine (i.e., video or live demonstration), which visually presents bodily information about how to execute the routine successfully. Such a learning strategy is known as action observation (AO), defined as the process of watching the actions of self or another individual *via* live demonstration or video with the intention of imitating the observed action (Eaves et al., 2016). There is a large body of evidence supporting the beneficial impact of AO for movement performance and learning across a range of contexts and populations; such as improved kinematics of surgical skills (Domuracki et al., 2015), greater futsal passing accuracy (Ghobadi et al., 2013), development and re-learning of fundamental movement patterns for individuals rehabilitating from stroke (Steel et al., 2018). Demonstrating the broad applicability of AO, studies have shown the benefits for the development of perceptual-cognitive skills, such as decision making in sports officials (Kittel et al., 2020). According to motor simulation theory (MST), the beneficial effects of AO are attributed to shared neural pathways with physical movement, referred to as functional equivalence (Jeannerod, 2001).

A key consideration for practitioners is deciding who the learner will observe (i.e., self or other person) and from which visual perspective (i.e., first or third-person). Aligning with MST, neuroscientific evidence indicates that observation of self may be more beneficial as it maximizes the neural overlap between observed and real movements (see Ste-Marie et al., 2020 for review). However, applied findings are inconsistent showing that both viewing of a skilled person and self improves performance outcomes (e.g., futsal pass; Ghobadi et al., 2013) and kinematics of complex skills (e.g., surgical techniques; Domuracki et al., 2015). Regarding visual perspective, previous research indicates that the type of skill being observed is a key consideration (Wright et al., 2022). A third-person perspective may be most appropriate for technical skills to adequately convey visual information on the skill technique (e.g., new gymnastics routine). Conversely, for outcome focused skills (e.g., putting in golf) a first-person view may be more appropriate (Kim et al., 2017).

The type of video footage implemented appears to be a key limitation for generating functionally equivalent AO interventions. Currently, AO interventions commonly utilize 2-D video recordings, potentially presenting users with less realistic practice environments (Vogt et al., 2013; Kim et al., 2017; Lindsay R. et al., 2022). An emerging technology known as 360°VR is a form of AO that could potentially address this issue (Kittel et al., 2019). 360°VR presents real-world video footage through a head-mounted display (HMD), allowing users to scan the performance environment, potentially increasing the realism of the training experience by providing visual information that is more representative of competitive experiences (Lindsay R. et al., 2022). Most notably, diagnostic of decision-making and anticipation skills have benefitted substantially from the innovative technologies such as 360°VR. Furthermore, the emergence of tools like 360°VR have increased the technological opportunities for specific decision-making practice in a range of sports, like basketball (Panchuk et al., 2018; Pagé et al., 2019), cricket (Discombe et al., 2022), and Australian Football umpires (Kittel et al., 2020). As outlined earlier, AO using 2-D footage has been shown to be an effective approach for developing motor skills in a broad range of technical skills (Ste-Marie et al., 2020). Emerging evidence indicates similar effects using 360°VR for developing technical skills and individual factors related to the production of motor skills in sport like improved motivation to learn in volleyball (Paraskevaidis and Fokides, 2020) and enhanced perceptual accuracy and knowledge in swimming (Roche et al., 2022). However, a distinguishing feature of 360°VR is the ability to generate more realistic and immersive learning environments. For example, Ferdig and Kosko (2020) observed improved immersion and perceptual capacity (i.e., attention) for teachers developing mathematical pedagogies. Taken together, these findings suggest that 360°VR may enhance the functional similarity between real-world movement and simulated action, presenting practitioners with the ability to design practice environments that accurately match simulated training with actual competitive environments (Jeannerod, 2001; Lindsay R. et al., 2022).

Grounded in key principles of ecological dynamics, a constraints-led approach (CLA) provides practioners with key considerations for creating practice environments that may facilitate the functional similarity between real-world and simulated settings, such as 360°VR. The CLA advocates the inclusion of key constraints in practice (e.g., variability of movement) to provide learners with opportunities for action (e.g., multiple passing options in response to defensive pressure) that are representative of competitive environments (Renshaw and Chow, 2019).

## 2. Ecological dynamics view of skill development

From an ecological dynamics perspective, skilled behavior is the result of continuous interactions between the learner and practice/performance environment (Button et al., 2020). Practitioners become designers of practice environments who encourage learners to explore and exploit functional relationships within the performance environment (Button et al., 2020) to better attune to opportunities for action and develop performance solutions matched to individual abilities, experiences, and skills (Renshaw and Chow, 2019). Subsequently, the focus of practice shifts from replicating a specific technique to facilitating learners in searching for individually appropriate movement solutions that align with individual constraints (e.g., height, weight, previous competition experiences). The term *skill adaption* has been proposed as an alternative to skill acquisition, defined as “enhancing one's functionality in a performance environment which can be continually improved.” (Renshaw and Chow, 2019). In support of this idea, Lindsay R. S. et al. (2022) found that novices who were prescribed an “ideal” technique to reproduce in acquiring a complex weightlifting movement actually demonstrated individualized movements, yet significantly improved overall performance. This indicates that reproducing “optimal” technique may not be a prerequisite for skilled behavior, rather the capacity to adapt and produce stable individualized performance solutions is critical (Button et al., 2020). Therefore, the aim of using 360°VR in developing skilled behavior may not necessarily be confined to reproducing expert technical models, but rather to facilitate exploration of individualized performance solutions (Renshaw and Chow, 2019; Chow et al., 2022). This exploration is likely to occur throughout skill adaptation as learners continue to explore individualized performance solutions to adapt to the performance environment, meaning that the use of 360°VR in developing skilled behavior may be applicable across stages of learning, from novices (e.g., Komar et al., 2019), amateur players (e.g., Discombe et al., 2022), as well as higher-performance athletes (e.g., Pagé et al., 2019).

## 3. Constraints-led approach (CLA) to 360°VR practice design

The CLA emphasizes that learning in sport and movement becomes a process of exploring, perceiving, and acting on relevant information sources that serve to guide movement. Subsequently, information in the performance environment regulates motor processes, which directly influence detection of information sources in the performance context, referred to as perception-action coupling (Gibson, 1979). Furthermore, an athlete's movement abilities will shape the opportunities for action perceived within a performance environment. For example, a hockey player may not perceive a shot on goal as the ball is on their weaker, reverse side. The demonstrated neural similarities between AO and actual movement suggest that 360°VR is capable of producing realistic training experiences that may enhance the link between an athletes abilities and environmental information from which action emerges. The inclusion of head movements using the HMD and scanning the environment allow for information to be perceived in a representative fashion, developing the link between information and movement (i.e., perception-action coupling). Therefore, using 360°VR learners can develop the ability to interpret relevant environmental information that is not available with 2-D flat-screen video (Lindsay R. et al., 2022). For example, evidence from educational contexts shows that 360°VR improve a teachers perceptual capacity (i.e., the amount of a situation that is visually available), which subsequently may increase their ability to “notice” and utilize key pieces of perceptual information within a given scenario (Ferdig and Kosko, 2020; Kosko et al., 2021). Applied in sport, 360°VR utilized by a batsmen in cricket may provide scanning capabilities that increase perceptual capacity and subsequently “notice” changes in fielding positions, which may then inform particular shot options.

Constraints are the parameters that facilitate a learner's self-organization processes and are categorized into three types: individual (e.g., physiological make-up, past experiences), task (e.g., scaling of equipment, number of learners involved in task), and environment (e.g., size of playing field) (Chow et al., 2022). Manipulating task constraints allows practitioners to invite specific movements from learners and guide learners to realize new opportunities for action that align unique individual experiences, development, and skills (Renshaw and Chow, 2019).

## 4. Practice design considerations for 360°VR

**Figure 1:**
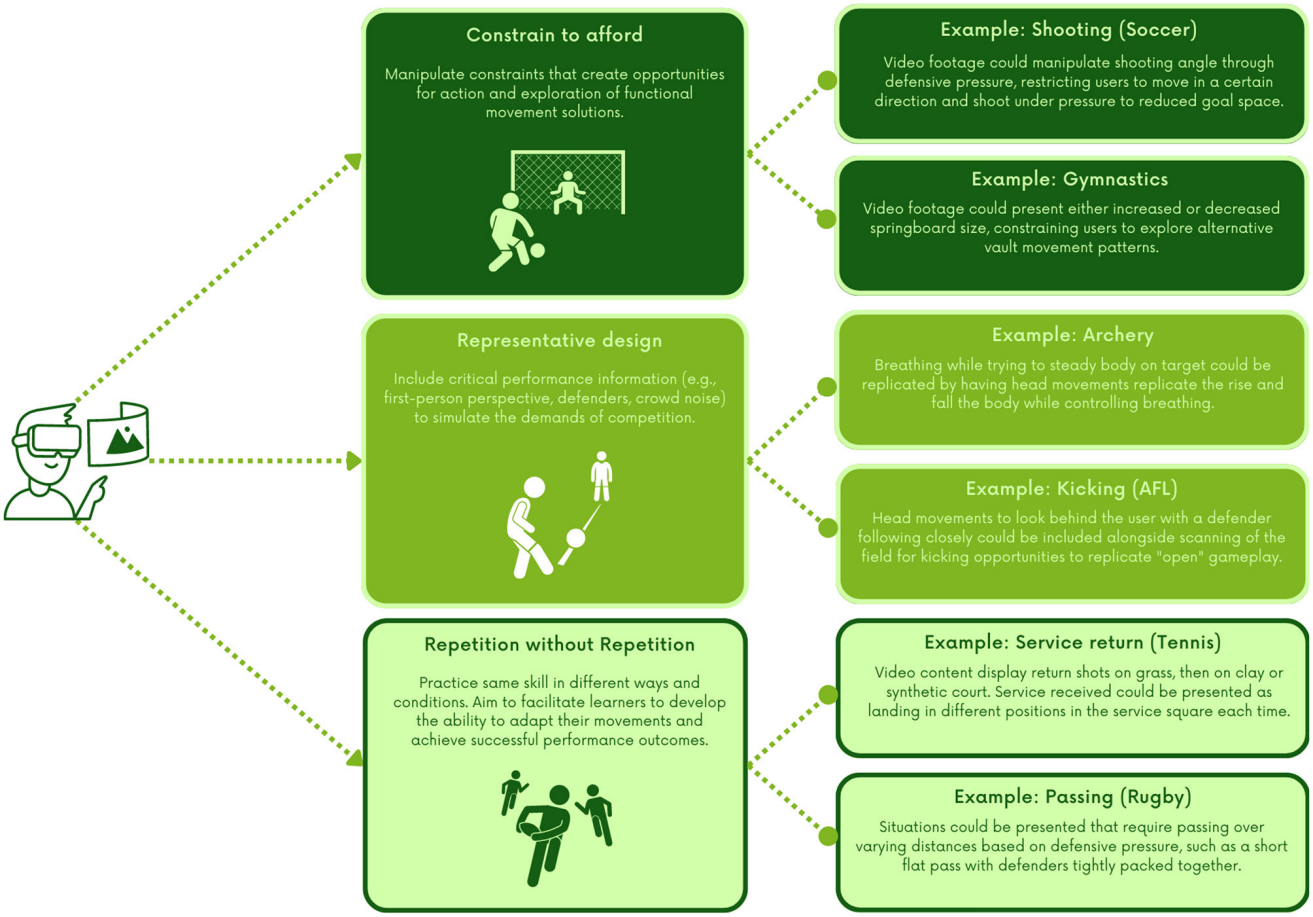
Practical applications for CLA to 360°VR practice design.

Using 360°VR as a form of AO to support skill development requires the practitioner to consider how practice will replicate critical elements of performance contexts to accurately match simulated and actual behaviors (i.e., functional equivalence). The CLA provides a number of key design principles that can support practitioners through this process which include: (1) constrain to afford—guide learners to opportunities for action to allow for exploration and exploitations of functional movement solutions through careful manipulation of task constraints; (2) representative design—simulate critical aspects of the performance environment in task design to provide key information to appropriately regulate movement; (3) repetition without repetition—ensure that skills are practiced with sufficient quantity but not in a repetitive way to amplify exploratory behavior in facilitating the development of individualized movement solutions (Correia et al., 2019; Chow et al., 2022). Figure 1 provides practical examples of how each environmental design principle can be applied in 360°VR.

### 4.1. Constrain to afford

From a CLA, movement variability, often referred to as exploration, is a key element of practice that can aid learners in attuning to various opportunities for action and developing movement solutions that align with their individual constraints (i.e., skill level, physiological make-up) (Komar et al., 2019). This contrasts with traditional skill acquisition approaches which may aim to reduce movement variability to produce consistent or “ideal” movement patterns (Spittle, 2021). Studies investigating 360°VR (e.g., Gänsluckner et al., 2017; Paraskevaidis and Fokides, 2020) and AO (Ste-Marie et al., 2020) appear to have often focused on providing participants with models of movement to replicate in videos, rather than exploring their own solutions. In CLA, practitioners can manipulate task constraints to amplify exploratory behavior of relevant affordances in the perceptual-motor landscape. When selecting the type of task constraints to use, practitioners have a number of options available, such reducing the distance between defending and attacking players in AFL (Correia et al., 2012). Applied to 360°VR, time to pass in soccer could be manipulated by recording video footage from a first-person perspective of passing from multiple distances from defending players (e.g., high direct pressure) that present a range of passing options with varying difficulties.

### 4.2. Representative design

Providing representative practice environments is a key component of a CLA for skill development in sport. Previous AO research has acknowledged the importance of presenting representative content, advocating for the use of footage from the first-person perspective for technical skills like golf-putting and dart-throwing (Kim et al., 2017; Wright et al., 2022). However, such research has primarily utilized 2-D video that emphasizes the replication of a prescribed movement pattern, where the same video footage of a skill is repeatedly watched by learners. The focus of AO from a CLA is to present a more representative practice environment—including variability of movement—to facilitate learners to develop adaptable and individualized movement solutions. Given the immersive nature of 360°VR, practitioners can capture highly representative content that accurately simulates relevant opportunities for action and potentially more authentic first-person perspective, facilitating a strengthening of perception-action coupling that underlie successful performance outcomes (Renshaw and Chow, 2019). For example, Panchuk et al. (2018) and Kittel et al. (2020) created 360°VR videos incorporating a first person-perspective immersed in the game play to create potentially more representative decision-making options. To check the representativeness of 360°VR content, practitioners should consider what perceptual information is presented and whether this information is reflective of actual performance, such as intensity and difficulty of the executed movement (see Figure 1 for example).

### 4.3. Repetition without repetition

Competitive environments are constantly changing, with learners very rarely executing skills in a similar fashion under the same conditions. The CLA advocates for practice that repeats the same skills regularly but not the same way every time, known as “repetition without repetition” (Renshaw and Chow, 2019). In applying this principle with 360°VR, a key consideration is the skill level of the learner. For higher-performing athletes, video footage could incorporate more complex task constraints that require technical skills to be executed in various different ways, such as changes in movement speed or distance. For example, for a high-level soccer player looking to develop their passing, 360°VR content could present passing options that randomly vary in distance, whereby the user scans to an open player which is covered by a defender requiring a longer passing option, followed by footage in which a short, quick pass is available (Oppici et al., 2018). For a skills in which different movement patterns can be utilized to achieve success (e.g., passing or dribbling in basketball) a first-person perspective may be most appropriate when using 360°VR as it most closely matches the visual information perceived during physical movement (Wright et al., 2022). Existing training studies in 360°VR in sport have created videos incorporating a first person-perspective with a range of variations in scenarios (e.g., Panchuk et al., 2018; Pagé et al., 2019; Kittel et al., 2020). For example, Kittel et al. (2020) utilized 125 and Pagé et al. (2019) 120 videos for decision making in training and Panchuk et al. (2018) used 56 scenarios which contained variations of 6 basic formations used by teams, possibly accommodating for “repetition without repetition”. The incorporation of scanning the environment from a first-person perspective is a real strength of 360°VR that may facilitate a more realistic user experience and maintaining of relevant perception-action couplings and the ability to adapt skill performance to changing performance conditions. As indicated earlier, there appear to be benefits from observing both the self and others and a range of skill levels in AO (Ste-Marie et al., 2020), and in line with the CLA advocated here, no matter the model, using AO in 360°VR that encourages exploration and attunement to key information sources guiding action.

## 5. Conclusion

This paper aimed to support practitioners in sport and movement settings in designing practice using 360°VR for skill acquisition and adaptation. We propose that 360°VR may be utilized as a form of AO to more accurately simulate critical aspects of performance contexts than traditional video modes. In line with the ecological dynamics perspective and CLA to skill acquisition, 360°VR may faciliate more representative practice design, as it allows users to scan the performance environment, potentially supporting perception-action coupling and helping performers to attune to relevant performance information. To achieve this, the CLA provides key practice design principles that practitioners can adapt to AO using 360°VR, including constrain to afford, representative design, and repetition without repetition. Practitioners are encouraged to consider adopting AO using 360°VR to support skill adaptation and acquisition and in doing so, consider these practical guidelines based on a CLA in sport and movement settings.

## Author contributions

RL and MS contributed to original conception and structure of this article. RL, SS, and MS contributed to writing the first draft of the manuscript. All authors were involved in manuscript revision, reading, and approval of the submitted version.
